# The Kinematic Relationship Between Two Spinal Regions and the Hip During Transitions From Standing to Sitting and Vice Versa

**DOI:** 10.1155/abb/5526457

**Published:** 2025-11-21

**Authors:** Raee S. Alqhtani

**Affiliations:** ^1^ Department of Rehabilitation Science, Physiotherapy Program, College of Applied Medical Science, Najran University, Najran, Saudi Arabia, nu.edu.sa

**Keywords:** lumbar, range of motion, sensors, thoracic, velocity

## Abstract

**Background:**

Previous studies have explored the kinematic relationship between the hip and lumbar spine during daily living activities. However, it is crucial to demonstrate the relationship between the hip, thoracic spine, and lumbar spine against hip kinematics during standing‐to‐sitting (SD‐to‐ST) and sitting‐to‐standing (ST‐to‐SD) tasks.

**Objectives:**

The study aimed to investigate the correlation between hip and thoracic kinematics, as well as hip and lumbar kinematics, during SD‐to‐ST and ST‐to‐SD tasks, and compare lumbar values with previous studies.

**Methods:**

A convenience‐based cohort study design was employed, and 29 males from the Najran University population were recruited (age = 30 years; mass = 73 kg). Double‐sided tape was used to attach four sensors to the spinous processes of T1, T12, and S1 and the side of the thigh in order to measure the range of motion (ROM) and velocity of the hip and two spinal regions during SD‐to‐ST and ST‐to‐SD.

**Results:**

The study found that hip ROM during SD‐to‐ST and ST‐to‐SD tasks was consistent at 64°, while thoracic and lumbar ROM were −1° and 48.75° for SD‐to‐ST and −10° and 47.81° for ST‐to‐SD, respectively. Hip velocity was similar at 62 and 65° s^−1^, and thoracic and lumbar velocities were 24 and 49.87° s^−1^ and 22 and 26.47° s^−1^, respectively.

**Interpretation:**

The study found no correlation between hip and thoracic spine in terms of ROM and velocity, and no correlation between regions. However, ROM and velocity significantly varied between regions. The lumbar spine outcomes were similar to previous research findings.

## 1. Background

Standing‐to‐sitting (SD‐to‐ST) and sitting‐to‐standing (ST‐to‐SD) movements are important activities of daily living (ADLs) that engage a number of lower limb and spine muscles [1]. Indeed, SD‐to‐ST and ST‐to‐SD activities are essential daily tasks that working people undertake roughly 60 times per day [2]. Professions like computer professionals, bankers, and desktop jobs require prolonged sitting postures, while security guards, professors, beauticians, and chemists require standing postures, as these occupations require different work environments and tasks [3]. Fundamental activities, including forward and backward bending, lifting objects, transitioning from SD‐to‐ST, and vice versa, have been consistently associated with spinal disorders, maybe arising from prolonged work hours without enough breaks [3]. Therefore, measuring spinal regions mechanically against hip region kinematics is important at a range of functional activities, including SD‐to‐ST and ST‐to‐SD tasks. At clinical assessment protocols, when clinicians or physiotherapists notice an impairment in range of motion (ROM), velocity, or movement behavior at the hip and spinal regions, it is indicative of the presence of spinal issues [4–6]. Indeed, understanding spinal region’s kinematics against hip kinematics can help clinicians and physiotherapists conduct accurate assessments and employ the most effective treatment methods [6, 7]. One of the essential indicators for assessing regional kinematics impairment is velocity, which is typically quantified in a graphical representation showing how joints move overtime [8–10]. Consequently, researchers have investigated the relationship between spinal and hip kinematics throughout the past 30 years. It was shown that problems in these areas could make daily life harder, which led to a study of hip‐related spinal regions [11]. Thus, the hip kinematics related to spinal regions are crucial for the successful evaluation and therapy of various spinal diseases [12]. Subsequently, substantial research has been conducted to reveal the correlation between the hip and various regions of the spine in both persons with lower back pain (LBP) and healthy subjects. Two studies [5, 13] have considered the relationship between ROM and angular velocity using what is known as the spatially associated ROM–angular velocity plot. These two studies established the basis and made it possible to measure movement behavior by calculating velocity in relation to movement time. Such kinematics information (ROM and velocity) can offer a distinct clinical picture of spinal dynamics [14]. The kinematics of SD‐to‐ST, ST‐to‐SD, or both tasks have been investigated in healthy populations [11, 15–18]. Alqhtani et al. [11] examined the correlation between the movement of the upper and lower segments of the lumbar region and hip motion during forward bending and other activities, such as transitioning from ST‐to‐SD and vice versa, excluding the thoracic spine from their analysis. A study conducted by Alqhtani et al. [15] examined the interaction between the upper and lower segments of the lumbar region and hip movement during daily activities, such as transitioning from ST‐to‐SD and vice versa, also discounting the thoracic spine from consideration. Leardini et al. [16] additionally assessed multisegment trunk kinematics during locomotion and basic exercises, encompassing SD‐to‐ST and ST‐to‐SD transitions, irrespective of hip involvement. Furthermore, Parkinson et al. [17] investigated the motion of the upper and lower segments of the lower back during the transition from SD‐to‐ST down, excluding hip and upper back movements, whereas Tully et al. [18] analyzed the movement of the spine and legs during the same transition. All participants’ samples who were employed in these studies were healthy individuals.

The lack of involvement of the thoracic area in everyday sagittal activities in these studies may be attributed to the characteristics of thoracic structures, such as ribs, which limit thoracic dynamics [19]. This view may diminish researchers’ interest in thoracic kinematics and their impact on movement behaviors. However, the thoracic region is not exempt from injury and impairment, and LBP and cervical spine pain have direct effects on thoracic ROM and velocity during the performance of daily tasks. Thus, identifying the relationship between hip and thoracic kinematics as well as lumbar and hip during SD‐to‐ST and ST‐to‐SD tasks can add new kinematic information that could be employed in spinal evaluation. To perform such measurements for clinical and research purposes, portable devices such as triaxial accelerometer sensors are necessary to capture ROM data overtime that describe multiple regions of the spine and hip [11]. While employing advanced instruments and various scenarios to measure hip–spinal correlation, particularly hip–lumbar spine kinematics, as examined by several authors, no studies have quantified the relationship between hip and thoracic kinematics, nor the correlation between hip and lumbar kinematics within the same protocol during SD‐to‐ST and vice versa tasks. Thus, the principal aim of the current study is to determine the relationship between hip and thoracic spine kinematics during transitions from SD‐to‐ST and ST‐to‐SD, in addition to comparing the lumbar spine with the hip in the same procedure, as well as to ascertain the degree of similarity between the study outcomes and previous research findings in the lumbar–hip region.

## 2. Methods

In this study, 29 males were recruited from the Najran University population (mean age = 30 years, standard deviation [St Dev.; SD] = 6 years; mean mass = 73 kg, SD = 13 kg). Neither of the participants had spinal or extremity disorders or neural conditions. This convenience‐based cohort study followed a similar protocol used by Alqhtani [20] by inviting participants from the Najran University population by email or direct verbal invitation to participate in this study in physiotherapy clinics at Najran University. When the participants agreed to participate, they received an information and consent form. Ethical approval for this study was obtained from the Najran University Ethics Committee for Scientific Research with reference number 010691‐023519‐DS.

**Figure 1:**
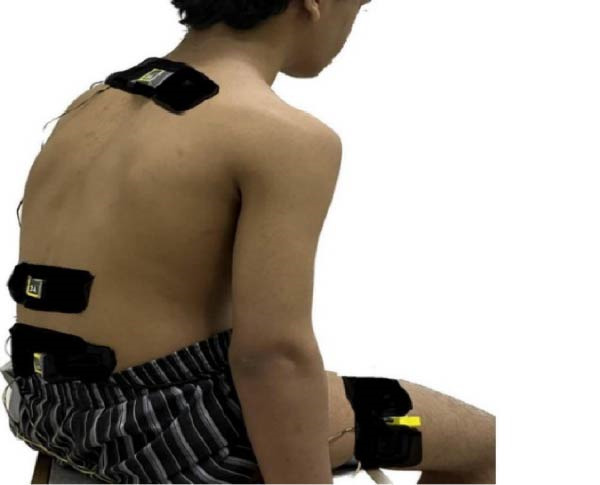
The scheme illustrates application 3A sensors on spinous processes of T1, T12, and S1 and on the lateral aspect of the thigh.

When the participants arrived at the experimental room, they were asked to read the written information sheet again and sign a consent form prior to data collection. The participant then moved to a private area to change clothes and wear a short pant. Warm‐up exercises including spinal forward and backward bending were introduced, prior sensors attaching to the participant’s body. The basic information on the safety and operation of the sensor system is explained to the participants individually. Essential information such as the age, height, and weight of the participant was written down. Four sensors were fixed with double‐sided tape over the spinous processes of T1, T12, S1, and on the middle of the lateral aspect of the thigh (Figure 1). Each sensor captured data on absolute orientation (tilt) with respect to gravity. In Figure 1, depicting the application of sensors on anatomical landmarks, the participant received an information and consent form; consequently, he completely consented to the publication of his photograph for research purposes and signed the following statement: “I consent to the publication of my body photograph, which displays indistinct facial features.”

To obtain the SD‐to‐ST kinematic, the participant starts the task from a standing position marked in front of a backless chair with their lower extremities touching the front of the chair to sit and perform the ST‐to‐SD kinematic; he should return from sitting to the start at standing position.

Data from hip, lumbar, and thoracic movement were gathered using a series of four accelerometers (triaxial accelerometers; THETAmetrix, Waterlooville, UK) coupled in a “daisy‐chain” arrangement, with each sensor measuring 24 mm^2^. Each accelerometer collected axial acceleration data for absolute orientation (tilt) with respect to gravity and was calibrated to record data at 30 Hz. This system has confirmed reliability for the analysis of spinal movement, when intraclass correlation coefficients ranged from 0.88 to 0.99 [19]. In the validation study of the triaxial accelerometer system, corroborated against a high‐precision rolly table as the gold standard by Alqhtani [20], the examination encompassed not only the sagittal and frontal planes (roll and pitch axes) but also assessed accuracy at 30° and 60° of roll crosstalk (with pitch fixed at 30° and subsequently at 60°) and at 30° and 60° of pitch crosstalk (with roll fixed initially at 30° and then at 60°). Correlations between the two measures at a confidence interval of 95%, yielded *r* = 0.99 across all axes, including roll and pitch crosstalk degrees, indicating that the triaxial accelerometer system is a valid instrument for monitoring spine movement in clinical environments. This pilot investigation has confirmed the system’s accuracy, demonstrating root mean square errors ranging from 0.70% to 1.39% when compared to a precision angle measuring table (THETAmetrix).

At this degree of accuracy in findings, corroborated by Alqhtani [20] during crosstalk trials, it is evident that the motions (SD‐to‐ST and ST‐to‐SD) are flawlessly accomplished in the sagittal plane and are unaffected by extraneous crosstalk movement.

**Figure 2:**
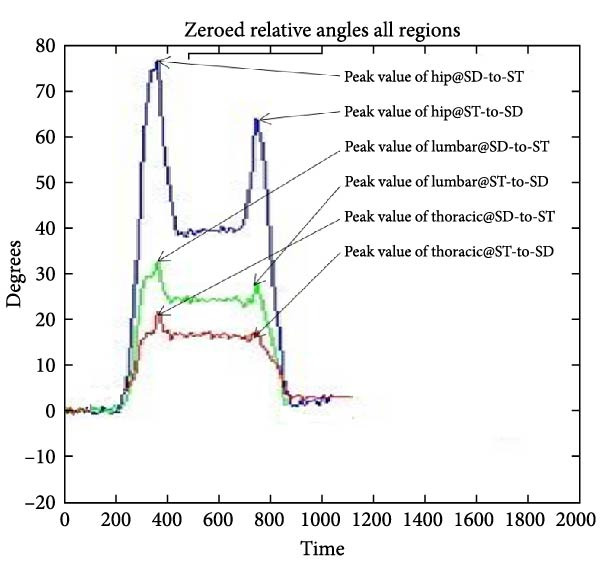
The MATLAB interface displays a participant’s thoracic spine and lumbar spine peak regional angulation motion in relation to the hip from SD‐to‐ST and from ST‐to‐SD.

Large amounts of data analysis require sophisticated software, so a series of MATLAB (MathWorks Inc., Natick, MA) software codes were written to create graphical figures depicting motion behavior and velocity overtime. This type of software can calculate the tilt angles with respect to gravity (absolute angles) for the ROM of a region and the relative kinematics between two adjacent distal and proximal sensors. The analysis involves two spinal regions (thoracic and lumbar) in connection to hip kinematic data utilizing MATLAB software. Such software enables the operator as well as the reader to observe and analyze the real‐time graphical representation of regional ROM and velocity, when data transmitted to MATLAB and filtered at 6 Hz (lowpass, Butterworth) to minimize high‐frequency noise [15]. Absolute angles for each sensor were determined in the sagittal plane relative to gravity. Thoracic spine kinematics was assessed by the relative angle between the T1 and T12 sensors, lumbar spine kinematics was derived from the relative angle between the T12 and S1 sensors, and hip kinematics was computed using the relative angle between the lateral thigh and S1 sensors (Figure 2). The movement analysis included two variables, ROM and velocity. In this study, the regional ROM was defined as the peak value of movement from the starting point to the endpoint at the complete stop point. Whereas positive velocity (+ve) was defined as a value of the rate change from the starting point of the task to the endpoint per unit of time. Negative velocity (−ve) was defined as the value of the rate change from the return point at the endpoint to the starting point of the task per unit of time.

The objective of the study was to examine the relationship between thoracic spine and hip kinematics, as well as lumbar–hip kinematics, within the same protocol. We obtained the study’s variables and variance using SPSS version 28 to identify any differences in the kinematics of the hip and spinal regions. The bivariate Pearson correlation is employed to calculate the correlation coefficient, denoted as *r*, which quantifies the degree of association between hip and thoracic kinematics as well as hip and lumbar kinematics. The classification of Evans [21] and Dancey and Reidy [22] has been utilized to ascertain the degree of correlation (*r*) among regional kinematics. The magnitude of the Pearson correlation coefficient, *r*, is categorized as extremely strong at 0.90–1.00 [21, 23], strong at 0.70–0.89 [21, 22], moderate at 0.40–0.69, weak at 0.10–0.39, and negligible from 0.00 to 0.09 [21].

## 3. Results

**Figure 3:**
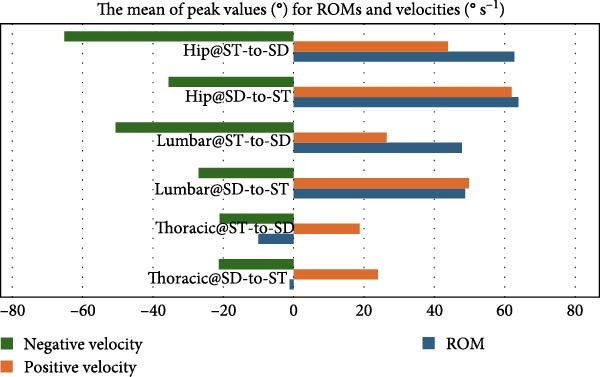
Displaying the mean of peak values (°) for ROMs and velocities during ST‐to‐ST and SD‐to‐ST tasks for thoracic, lumbar and hip kinematics.

Tables 1–3 and Figure 3 show the average maximum values (in degrees) for thoracic, lumbar, and hip ROMs and velocities during SD‐to‐ST and ST‐to‐SD transitions. Table 1 shows a difference in thoracic region ROM (−1.16° from SD‐to‐ST and −10° from ST‐to‐SD), while Table 2 shows slightly closer positive velocities (23.97 and 18.78° s^−1^) and Table 3 shows similar negative velocities (−21.28 and −21.59° s^−1^). The lumbar ROM during the transition from SD‐to‐ST and from ST‐to‐SD exhibited almost equal values in Table 1 (48.75° and 47.82°, respectively), whereas the lumbar positive velocities for SD‐to‐ST and from ST‐to‐SD were 49.87 and 26.47° s^−1^, respectively, as shown in Table 2. In contrast, the lumbar negative velocities were −27.66 and −50.61° s^−1^, respectively (Table 3). The hip ROM during the transition from SD‐to‐ST and from ST‐to‐SD exhibited almost equal measurements (62° and 62.8°, respectively) as shown in Table 1. Meanwhile, the hip positive velocities for the transitions from SD‐to‐ST and ST‐to‐SD were recorded at 62 and 43.8° s^−1^, respectively (Table 3). The hip negative velocities recorded were −35.56 and −65.12° s^−1^, respectively, in Table 3, indicating a velocity magnitude that was twice during ST‐to‐SD compared to SD‐to‐ST. The positive velocity values of the thoracic spine were roughly 50% lower than the positive velocity values of the hip during the two tasks, as well as the lumbar positive velocity at ST‐to‐SD, which was 50% lower than the hip but similar to the positive velocity at the SD‐to‐ST task.

**Table 1:** Demonstrating ROM means (°), correlation (*r*), and significant differences (*p*‐value) for ROM of thoracic, lumbar, and hip regions during SD‐to‐ST and ST‐to‐SD tasks.

Correlations							
		Tx ROM@SD‐to‐ST	Lx ROM@SD‐to‐ST	Hx ROM@SD‐to‐ST	Tx ROM@ST‐to‐SD	Lx ROM@ST‐to‐SD	Hx ROM@ST‐to‐SD
Regional ROM means (St Dev.)		−1.16 (11.69)	48.75 (22.15)	63.91 (19.67)	−10 (14)	47.81 (16.91)	62.81 (21.27)

Tx ROM@SD‐to‐ST	Pearson correlation (*r*)	1	−0.033	**0.078**	−0.222	−0.157	0.295
	Sig. (*p*‐value)	—	0.865	**0.689**	0.247	0.415	0.120
Lx ROM@SD‐to‐ST	Pearson correlation (*r*)	−0.033	1	**−0.206**	−0.118	0.272	−0.057
	Sig. (*p*‐value)	0.865	—	**0.285**	0.542	0.153	0.768
Tx ROM@ST‐to‐SD	Pearson correlation (*r*)	−0.222	−0.118	−0.151	1	−0.022	**0.151**
	Sig. (*p*‐value)	0.247	0.542	0.435	—	0.909	**0.434**
Lx ROM@ST‐to‐SD	Pearson correlation (*r*)	−0.157	0.272	0.024	−0.022	1	**−0.379**
	Sig. (*p*‐value)	0.415	0.153	0.900	0.909	—	**0.043**

*Note:* The bold values show Pearson *r* and *p*‐values for Tx–Hx and Lx–Hx ROM correlations during SD‐to‐ST and ST‐to‐SD, highlighting the study’s key kinematic focus and lumbar comparison.

Abbreviations: Hx, hip; Lx, lumbar; ROM, range of motion; SD‐to‐ST, standing to sitting; St Dev., standard deviation; ST‐to‐SD, sitting to standing; Tx, thoracic.

**Table 2:** Demonstrating positive velocity (° s^−1^), correlation (*r*), and significant differences (*p*‐value) for positive (+v) velocity of thoracic, lumbar, and hip regions during SD‐to‐ST and ST‐to‐SD tasks.

Correlations							
		Tx +Vel@SD‐to‐ST	Lx +Vel@SD‐to‐ST	Hx +Vel@SD‐to‐ST	Tx +Vel@ST‐to‐SD	Lx +Vel@ST‐to‐SD	Hx +Vel@ST‐to‐SD
Regional +velocity means (St Dev.)		23.97 (13.99)	49.87 (23.14)	62 (21.36)	18.78 (9.64)	26.47 (16.41)	43.84 (26.14)

Tx +Vel@SD‐to‐ST	Pearson correlation (*r*)	1	0.653	**−0.031**	−0.093	0.433	−0.275
	Sig. (*p*‐value)	—	0.000	**0.874**	0.631	0.019	0.149
Lx +Vel@SD‐to‐ST	Pearson correlation (*r*)	0.653	1	**0.109**	−0.130	0.243	−0.109
	Sig. (*p*‐value)	0.000	—	**0.575**	0.503	0.205	0.575
Tx +Vel@ST‐to‐SD	Pearson correlation (*r*)	−0.093	−0.130	−0.133	1	−0.244	**0.218**
	Sig. (*p*‐value)	0.631	0.503	0.490	—	0.203	**0.256**
Lx +Vel@ST‐to‐SD	Pearson correlation (*r*)	0.433	0.243	−0.025	−0.244	1	**−0.672**
	Sig. (*p*‐value)	0.019	0.205	0.896	0.203	—	**0.000**

*Note*: The bold values show Pearson *r* and *p*‐values for Tx–Hx and Lx–Hx positive velocity correlations during SD‐to‐ST and ST‐to‐SD, highlighting the study’s key kinematic focus and lumbar comparison.

Abbreviation: +Vel., positive velocity.

**Table 3:** Demonstrating negative velocity (° s^−1^), correlation (*r*), and significant differences (*p*‐value) for negative (−v) velocity of thoracic, lumbar, and hip regions during SD‐to‐ST and ST‐to‐SD tasks.

Correlations							
		Tx −Vel@SD‐to‐ST	Lx −Vel@SD‐to‐ST	Hx −Vel@SD‐to‐ST	Tx −Vel@ST‐to‐SD	Lx −Vel@ST‐to‐SD	Hx −Vel@ST‐to‐SD
Regional −velocity means and (St Dev.)		−21.28 (10.70)	−27.66 (13.44)	−35.56 (17.51)	−21.59 (15.27)	−50.61 (23.19)	−65.12 (30.55)

Tx −Vel@SD‐to‐ST	Pearson correlation (*r*)	1	0.397	**−0.076**	−0.064	0.259	0.015
	Sig. (*p*‐value)	—	0.033	**0.694**	0.743	0.175	0.938
Lx −Vel@SD‐to‐ST	Pearson correlation (*r*)	0.397	1	**−0.147**	0.153	0.286	−0.025
	Sig. (*p*‐value)	0.033	—	**0.446**	0.428	0.132	0.897
Tx −Vel@ST‐to‐SD	Pearson correlation (*r*)	−0.064	0.153	−0.300	1	0.355	**−0.003**
	Sig. (*p*‐value)	0.743	0.428	0.114	—	0.059	**0.988**
Lx −Vel@ST‐to‐SD	Pearson correlation (*r*)	0.259	0.286	0.147	0.355	1	**0.093**
	Sig. (*p*‐value)	0.175	0.132	0.446	0.059	—	**0.631**

*Note:* The bold values show Pearson *r* and *p*‐values for Tx–Hx and Lx–Hx negative velocity correlations during SD‐to‐ST and ST‐to‐SD, highlighting the study’s key kinematic focus and lumbar comparison.

Abbreviation: −Vel., negative velocity.

Tables 1–3 demonstrated a correlation and a substantial difference: thoracic and hip ROM at SD‐to‐ST exhibited a negligible correlation (*r* = 0.078) and a significant difference (*p* = 0.689). Thoracic positive velocity at SD‐to‐ST was not correlated with hip positive velocity (*r* = −0.031, *p* = 0.874). A negligible correlation (*r* = −0.076, −0.003, and 0.093) was seen between thoracic negative velocity at SD‐to‐ST and hip negative velocity, as well as between lumbar negative velocity and hip negative velocity at ST‐to‐SD, with statistically significant differences (*p* = 0.694, 0.988, and 0.631). The SD‐to‐ST task shown a weak association (*r* = 0.151 and −0.379) between hip and thoracic as well as lumbar ROM. Although lumbar and hip ROM showed no significant difference, thoracic and hip ROM did exhibit a difference (*p* = 0.689). The lumbar ROM was not substantially different (*p* = 0.043). In the SD‐to‐ST task, lumbar ROM and hip ROM exhibited a weak connection (*r* = −0.206) and a statistically significant difference (*p* = 0.285). Hip ROM exhibited a weak correlation (*r* = −0.109), but the lumbar positive velocity from SD to ST demonstrated a statistically significant difference (*p* = 0.575). The positive velocity of the thoracic and lumbar regions at ST‐to‐SD differed from that of the hip (*p* = 0.256), exhibiting a poor correlation (*r* = −0.218) and a moderate correlation (*r* = −0.67). This task demonstrated no substantial difference between the lumbar and hip areas (*p* = 0.000). The negative velocity of the thoracic and lumbar regions at SD‐to‐ST was significantly different from the negative velocity of the hip (*p* = 0.694 and 0.446, respectively), exhibiting a weak association (*r* = −0.076 and −0.147).

Figure 3 also provides a clear and direct picture of the ROM and regional velocity. The ROM is large in both tasks, as is the positive velocity when sitting from a standing position. However, this velocity is approximately 20° s^−1^ lower when standing from sitting, in contrast to the negative velocity, which appears large in the transition from SD‐to‐ST.

## 4. Discussion

This study aimed to investigate the relationship between hip and thoracic spine kinematics during transitions from SD‐to‐ST and ST‐to‐SD, while also comparing the lumbar spine with the hip within the same procedure and determining the degree of congruence between the study results and prior research findings in the lumbar–hip region. The present research effectively showcased a number of regional results that contributed fresh insights to the literature. First, it illustrates the correlation between hip and thoracic spine kinematics during transitions from sitting down to standing and SD‐to‐ST down. Second, evaluate the extent of congruence between the study results and prior research findings in the lumbar–hip area. The ROM for thoracic motions for two tasks was −1.16° for the SD‐to‐ST task and −10° for the ST‐to‐SD task. The negative results revealed that the trunk shifted posteriorly, exhibiting more posterior movement during the transition from ST‐to‐SD, whereas the hip motions measured 63.91° and 62°, respectively, which significantly diverge from the thoracic movements in these two tasks. So far, there has been no information about thoracic ROM in comparison to hip ROM during these tasks. Nevertheless, hip ranges of motion for these activities were nearly identical to the approximately 64° observed in study by Alqhtani et al. [11], Shum et al. [13],and Coghlin and McFadyen [24], hence affirming the validity of the measuring apparatus and scenario application. Thoracic positive velocity values were about one‐third of hip positive velocity during SD‐to‐ST, measuring 23.97° s^−1^ compared to hip positive velocity at 63.9° s^−1^, and during ST‐to‐SD, measuring 18.78° s^−1^ versus hip positive velocity at 43.84° s^−1^. Nonetheless, thoracic velocity measurements for the two activities were comparable (SD‐to‐ST = −21.28° s^−1^ and ST‐to‐SD = −21.59° s^−1^) in contrast to the hip’s negative velocities of −35.56 and −65.12° s^−1^. Thoracic negative velocity showed nearly 50% of hip velocity values during SD‐to‐ST. As previously mentioned, prior research lacked thoracic kinematic data for comparison with the hip during both activities; nonetheless, the negative velocities of the hip were around −35 and −64.3, as reported by Alqhtani et al. [11]. The study’s findings at the lumbar spine demonstrated consistency in ROM throughout both activities (48.75° and 47.81°), with positive velocities (49.87 and 26.47° s^−1^) and negative velocities (−27.66 and −50.61° s^−1^), respectively. The current study’s findings supported those by Alqhtani et al. [11] and Shum et al. [13], who demonstrated similar lumbar ROM as well as positive and negative velocities. The substantial St Dev., varying from 9.6° to 30.5° across all regional kinematic tasks, may be attributed to several factors, including participants’ ages, biological characteristics and the individual variability in task transitions influenced by distinct movement styles, as there was no standardization of transitions in the study protocol.

The study found no correlation (*r* = 0.078) between thoracic and hip ROM at SD‐to‐ST and no association (*r* = −0.031) between thoracic and hip positive velocity. Also, thoracic, hip, and lumbar negative velocity at SD‐to‐ST had negligible correlations (*r* = −0.076, −0.003, and 0.093). This study did not examine thoracic, lumbar, and hip angulation velocities in a healthy population while performing these tasks, so it cannot compare regional positive and negative velocities to previous research. ROM has shown only a weak to moderate link with the lumbar and hip movement needed for specific functional tasks in ADLs, such as SD‐to‐ST and ST‐to‐SD tasks [11]. Consequently, the weak to moderate correlations identified between regional ROM and velocities across two tasks in this study ranged from *r* = 0.109 to *r* = 0.67, aligning with the findings of [11].

This study is the inaugural investigation of the kinematic relationship between the hip and thoracic regions, as well as between the hip and lumbar regions, utilizing a consistent protocol for two prevalent daily tasks through wearable sensor technology. This study is distinctive yet the findings on hip kinematics are corroborated by prior research examining hip‐lumbar relationships during ST‐to‐SD and SD‐to‐ST tasks [11, 25]. The current study’s hip velocity exceeded previous findings by roughly 7° s^−1^ during SD‐to‐ST and 12° s^−1^ during ST‐to‐SD than previous findings. A few studies [18, 26] have reported more contributions at the hip joint ROM and velocity during ST‐to‐SD than those found in this study. Differences in previous studies protocols, measurement devices, participant ages, and participant health status are factors that may have caused the disparate findings of these studies. However, examining the hip kinematics in relation to the upper and lower lumbar regions during both tasks and discovered findings that closely align with hip kinematics in the present study.

It was not feasible to compare the findings of the current study regarding the hip and thoracic regions with previous studies, as none of them examined the relationship between these two regions during the two tasks. The findings may provide a normative reference for spinal measurements in healthy individuals and contribute new insights to the field of spinal biomechanics research. Consequently, clinicians should refrain from implementing this study procedure in clinical assessments due to the lack of kinematic correlations between the hip and thoracic regions during SD‐to‐ST and ST‐to‐SD tasks. The application of sensor technology to monitor movement behaviors overtime may enable researchers and practitioners to integrate these devices into clinical trials for spine evaluation.

This study was limited by a few challenges, including the gender of the participants (only men) and the age of the participants (ranging from 18 to 46 years) and the application of sensors. As the sample represented a healthy population of young men of different ages, any findings relating to the elderly, healthy women, or those with spinal pain may differ from the present findings. The study protocol requiring sensor application directly on the skin caused women to deny participating in the current study, which required the use of a single‐sex (male) sample. Moreover, the variety between participants’ ages could affect the data, as younger populations have higher levels of flexibility than older populations, which may lead to differences in ROM, velocity, and quality of movement. Future work will be necessary to include both sexes (men and women) and different age groups, as well as compare healthy and symptomatic individuals.

In conclusion, the findings of this study indicate that there is no relationship between hip and thoracic spine kinematics during SD‐to‐ST and ST‐to‐SD tasks; however, the findings could add useful normative information to clinical evaluation practices. Comparing the lumbar spine with the hip in the same procedure, provided similarity between this study outcome and previous research findings in the lumbar‐hip region. Although there are substantial differences between hip and thoracic spine ROM and velocity, physiotherapists and orthopedists should consider when performing clinical assessments that the contribution of the thoracic spine is minor during SD‐to‐ST and ST‐to‐SD tasks, implying that thoracic kinematic disorders during these tasks are very rare.

## Conflicts of Interest

The author declares no conflicts of interest.

## Funding

The author is thankful to the Deanship of Graduate Studies and Scientific Research at Najran University for funding this work under the Easy Funding Program (Grant NU/EFP/MRC/13/214).

## Data Availability

The data that support the findings of this study are available from the corresponding author upon reasonable request.
